# A Rare Oncologic Emergency: Spontaneous Tumor Lysis Syndrome in Metastatic Colon Adenocarcinoma

**DOI:** 10.5811/cpcem.2019.9.43770

**Published:** 2019-10-14

**Authors:** Kathleen E. Kalmbach, Leena T. Rahmat, Justyna A. Wos, Nicholas J. Daniel

**Affiliations:** * University of Massachusetts Medical School, Baystate Medical Center, Department of Emergency Medicine, Springfield, Massachusetts; † School of Education, Human and Health Sciences: Physician Assistant Studies, Bay Path University, Longmeadow, Massachusetts; ‡ Johns Hopkins University, Sidney Kimmel Comprehensive Cancer Center, Department of Oncology, Washington, District of Columbia; § Springfield College, Department of Physician Assistant Studies, Springfield, Massachusetts

## Abstract

Tumor lysis syndrome is an oncologic emergency that can present with variable symptoms and is truly a laboratory-based diagnosis without pathognomonic clinical findings. The classical teaching is to consider this diagnosis in cancer patients undergoing chemotherapy. We present the case of a 66-year-old female with newly diagnosed metastatic liver adenocarcinoma, not on chemotherapy, who was diagnosed with spontaneous tumor lysis syndrome. Cognizance of this syndrome and associated laboratory findings are paramount to diagnosis and rapid intervention.

## INTRODUCTION

Tumor lysis syndrome (TLS) is an oncologic emergency seen in patients with rapidly proliferating hematologic malignancies following exposure to systemic chemotherapy. It is a metabolic disorder characterized by hyperuricemia, hyperkalemia, hypocalcemia, and hyperphosphatemia, which can result in acute kidney injury, cardiac arrhythmias, and central nervous system toxicity. Spontaneous TLS (STLS) is rare and occurs in the absence of cytotoxic therapy. It is characterized by spontaneous cellular death in rapidly proliferating malignancies. STLS in patients with solid cancers is associated with a high mortality.^1^ We present a case of a woman recently diagnosed with metastatic colon adenocarcinoma who presented with STLS.

## CASE REPORT

A 66-year-old female with past medical history of hypertension, hyperlipidemia, and diabetes mellitus presented to the emergency department (ED) with subacute weakness and dyspnea. Five weeks prior to this presentation, she was seen in the ED for abdominal pain and dyspnea and was found to have large, bilateral pleural effusions and anemia. Extensive further work-up revealed a diagnosis of stage IV, poorly differentiated adenocarcinoma of the transverse colon. Imaging revealed diffuse large lymphadenopathy throughout the abdomen, retroperitoneum, and mediastinum with multiple pulmonary nodules. She underwent an open extended right hemicolectomy with ileostomy as well as resection of the jejunum due to tumor invasion. Thoracentesis was performed and she was eventually discharged home to follow up with oncology for discussions about immunotherapy or chemotherapy.

At the current ED visit, the patient’s vitals were as follows: blood pressure 121/39 millimeters of mercury, pulse rate of 88 beats per minute, respiratory rate of 20 breaths per minute, and an oxygen saturation of 98% on three liters of oxygen via nasal cannula. Physical examination was pertinent for an ill-appearing female with mild jaundice, abdominal distention with mild tenderness, and decreased breath sounds with rales in the bilateral lower lung fields. A chest radiograph showed bilateral pleural effusions. The complete blood count was unremarkable. The complete metabolic panel demonstrated hyponatremia, hyperkalemia, uremia, acute kidney injury, and transaminitis (Table). Given the new acute kidney injury and hyperkalemia, there was suspicion for STLS. Additional diagnostics were obtained and revealed hypocalcemia, hypermagnesemia, hyperphosphatemia, and hyperuricemia (Table). Electrocardiogram demonstrated minimal changes in T-wave morphology as well as a slight increase in QRS duration. Review of the patient’s chart showed that all of the laboratory abnormalities were new in comparison to four weeks prior.

A diagnosis of STLS was made in this patient with metastatic, poorly differentiated adenocarcinoma, who had not been initiated on systemic chemotherapy. Intravenous crystalloids, rasburicase, and treatment for acute hyperkalemia with calcium gluconate, insulin, dextrose, kayexalate, and sodium bicarbonate were initiated. After consulting the renal and oncology teams, the patient was admitted. Given the poor prognosis, the patient declined dialysis and further invasive measures. Her status was changed to comfort measures only and she died two days later.

## DISCUSSION

TLS can be therapy-related (cytotoxic therapy) or spontaneous (no exposure to cytotoxic therapy). TLS is more commonly seen in patients with hematologic malignancies compared to solid malignancies, due to increased proliferation rates and sensitivity to cytotoxic therapy.^1^–^4^ Solid tumors with a large tumor burden are at increased risk of TLS. STLS, which occurs in the absence of cytotoxic therapy, is more rare and most often seen in hematologic malignancies with high proliferation rates, such as acute leukemia and aggressive lymphomas, such as Burkitt lymphoma.

One retrospective review found that STLS occurred in 1.1% of patients with hematologic malignancy and acute renal failure.^4^ The rate of STLS in patients with solid malignancies is even lower, with the true prevalence remaining uncertain due to a paucity of literature comprised mainly of case reports.^4^ It has been described in patients with metastatic and advanced malignancies including small cell lung cancer, cholangiocarcinoma, hepatocellular carcinoma, and only two other cases of colon adenocarcinoma.^1^,^3^–^5^ The laboratory abnormalities outlined in the Cairo-Bishop definition of TLS include hyperuricemia, hyperkalemia, hyperphosphatemia, and hypocalcemia, either outside the reference range or a 25% change from baseline.^2^ Potential complications of these metabolic abnormalities include acute kidney injury, cardiac arrhythmias, and seizures.^1^–^4^

There is a 20–50% mortality rate in TLS occurring in solid tumors if undiagnosed, or diagnosed too late.^6^ Spontaneous development of TLS in solid tumors reflects large tumor burden and likely portends an even higher mortality. Timely recognition and intervention following a diagnosis of STLS is vital. Management consists of intravenous hydration, correction of electrolyte abnormalities, and treatment of hyperuricemia, which can lead to obstructive uropathy. Allopurinol can be used prophylactically in patients at high risk for TLS, but should not be used for hyperuricemia in acute TLS.^5^ Acute hyperuricemia in TLS or STLS can be treated with rasburicase, a recombinant urate oxidase enzyme that catalyzes uric acid to allantoin, or hemodialysis. Patients should have strict monitoring of urine output as persistent oliguria may be an additional indication for hemodialysis, along with hyperkalemia and hyperuricemia.

**CPC-EM Capsule**

What do we already know about this clinical entity?

*Tumor lysis syndrome (TLS) is an oncologic emergency identified by lab abnormalities: hyperkalemia, hyperphosphatemia, hyperuricemia, hypocalcemia, and acute kidney injury*.

What makes this presentation of disease reportable?

*TLS may occur spontaneously, but typically it occurs in the setting of hematologic malignancies. Spontaneous TLS in solid tumors is rare*.

What is the major learning point?

*The classic lab abnormalities of TLS should prompt consideration of this complication in any oncology patient, regardless of concomitant chemotherapy*.

How might this improve emergency medicine practice?

*Early recognizance of TLS can lead to rapid management, mitigation of complications, and improved emergency medical care*.

Our patient had been recently diagnosed with metastatic colon adenocarcinoma with diffuse metastases. We believe the heavy tumor burden and rapid proliferative rate increased her risk of developing STLS.

## CONCLUSION

Consideration of a diagnosis of TLS was historically reserved for patients with the classic lab abnormalities who were undergoing chemotherapy. Reports of STLS in both hematologic and solid malignancies are increasing in the literature. Many of these patients will first present to the ED and it is pertinent for the emergency physician to recognize this oncologic emergency. Striking electrolyte imbalances and acute kidney injury in any cancer patient ought to prompt consideration of STLS, as the associated complications can be life-threatening.

## Figures and Tables

**Table:** Laboratory values of patient with tumor lysis syndrome.

Component	Value	Reference range
White blood cells	9.7	4.0–11.0 k/mm^3^
Hemoglobin	11.4	11.7–15.5 Gm/dL
Hematocrit	36.9	35.7–45.8%
Platelet count	473	150–460 k/mm^3^
INR	1.2	0.9–1.1
Prothrombin time	12.6	9.7–12.2 seconds
Sodium	123	133–145 mmol/L
*Potassium	7.7	3.6–5.2 mmol/L
Chloride	87	98–107 mmol/L
Bicarbonate	16	22–29 mmol/L
Anion gap	20	4–17
Glucose	137	70–99 mg/dL (fasting)
BUN	79	8–23 mg/dL
Creatinine	6.4	0.5–1.0 mg/dL
*Calcium, Ionized	1.02	1.13–1.32 mmol/L
Magnesium	2.4	1.3–1.9 mEq/L
LDH	544	94–250 units/L
Alkaline phosphatase	882	35–104 units/L
AST	81	0–32 units/L
ALT	36	0–33 units/L
Bilirubin, total	5.9	0–1.2 mg/dL
Lactate	1.7	0.5–2.2 mmol/L
Troponin T Quant	<0.01	0.0–0.09 ng/mL
Nt-ProBNP	3,029	0–125 pg/mL
Haptoglobin	334	30–200 mg/dL
*Uric acid	23.9	1.6–7.6 mg/dL
*Phosphorus	10.1	2.5–4.5 mg/dL

* Laboratory abnormalities listed in the Cairo-Bishop definition of tumor lysis syndrome (*TLS*). Creatinine ≥1.5× upper limit of normal meets criteria for clinical TLS, along with the defined laboratory changes (2).

*k*, kilogram; *mm^3^*, cubic millimeter; *gm*, gram; *dL*, deciliter; *INR*, international normalized ratio; *mmol*, millimole; *L*, liter; *mg*, milligram; *BUN*, blood urea nitrogen; *mEq*, milliequivalent; *LDH*, lactate dehydrogenase; *AST*, aspartate aminotransferase; *ALT*, alanine aminotransferase; *ng*, nanogram; *mL*, milliliter; *pg*, picogram.
